# Charting the knowledge landscape of mathematics learning disabilities: an analysis of intellectual structure and research frontiers

**DOI:** 10.3389/fpsyg.2025.1652056

**Published:** 2025-10-21

**Authors:** Chen Li, Lin Wang

**Affiliations:** School of Education, Taiyuan Normal University, Jinzhong, China

**Keywords:** mathematics learning disabilities, visual analytics, knowledge mapping, trends, frontiers

## Abstract

Mathematical Learning Disabilities (MLD) impact 5–10% of school-age children globally, with associated academic challenges and mental health risks garnering significant societal concern. Investigating scholarly work in this domain enhances our understanding of the mechanisms underlying MLD, informing early intervention strategies. Based on Web of Science data, this study employed CiteSpace for bibliometric visualization analysis of MLD literature, revealing that: (1) Seven European and American scholars form a core group of highly cited authors, and ten foundational papers establishing the field’s theoretical framework; (2)The hotspots of research focus on cognitive mechanisms of mathematical disorders, brain-neurological mechanisms, socio-cultural factors, educational strategies; (3)Developmental trajectories demonstrate a cyclical pattern of “theory construction-practice verification-theory optimization.” Future MLD research will evolve towards precise diagnosis and comprehensive systematic intervention.

## 1 Introduction

Since Samuel Kirk pioneered the concept of “learning disabilities” in 1963, research on learning disabilities has garnered attention from the medical, educational, and psychological fields. As a significant subtype of learning disabilities, Mathematical Learning Disabilities (MLD) have gradually emerged as a central focus in special education research, due to their intricate cognitive-neurological mechanisms and profound socio-adaptive impacts.

As defined by the Diagnostic and Statistical Manual of Mental Disorders (DSM-V), MLD refer to specific deficits in core quantitative processing, mathematical computation, and logical reasoning domains among individuals of normal intelligence. These persistent difficulties, resistant to improvement through standard educational strategies, lead to mathematical abilities substantially below age-appropriate expectations (McGuire, 2015). Research indicates that MLD affect 5–10% of school-age children globally (Bartelet et al., 2014), with the academic problems, occupational constraints, and mental health risks becoming a challenge to public safety. In the past 5 years, with the deep intersection of cognitive neuroscience, educational technology and psychometrics, MLD research has made breakthroughs in heterogeneity theory construction(Munez et al., 2023), technology-enhanced interventions (Islim et al., 2024; Satsangi and Raines, 2023) and co-morbidity mechanism resolution (Payne and Yoon, 2024; Wakeman et al., 2023).

Knowledge mapping technology, with its powerful multi-source data processing and visual presentation capabilities, has demonstrated its value in the field of education and psychology research (Li and Chen, 2022). By constructing conceptual networks and co-occurrence clustering, the method is able to accurately identify the knowledge base of the domain, track the path of hotspot changes, explore the development of the frontier.

Through a literature search, we found no articles that directly applied bibliometric methods to study MLD. However, several recent studies in related areas have provided highly relevant insights. Espina et al. (2022) used bibliometrics to review the field of dyscalculia, revealing its overall development trends. Mutlu and Polat (2024) conducted a comprehensive bibliometric and content analysis of dyscalculia research using the Web of Science database, aiming to identify key themes, research trends, and knowledge gaps in the field. Their study found that terms such as dyscalculia, mathematical difficulties, dyslexia, and developmental dyscalculia were the most frequent keywords. It also noted that highly cited articles often focused on sample characteristics and methodologies, while emphasizing the importance of interdisciplinary collaboration in understanding the nature and causes of mathematical learning disabilities. Similarly, Deda et al. (2024) performed a bibliometric analysis based on the Scopus database (2017–2022), research topics in dyscalculia mainly focused on *students with deficits*, *difficulties in addition and subtraction*, *mathematical difficulties,* and *teaching methods in primary school*. Meanwhile Drljić and Doz (2025) applied the method to explore the use of digital tools in supporting students aged 12–18 with mathematical learning difficulties. However, neither of these articles provides a comprehensive and systematic literature analysis specifically focused on MLD.

Therefore, this study systematically collected MLD-related literature from the Web of Science Core Collection database (2000–2024), conducted scientometric analysis using CiteSpace to obtain the current research status, reveal hot topics, research frontiers, and evolutionary trajectories in this field, as well as providing literature support for further theoretical exploration and the construction of multidimensional intervention systems for MLD.

## 2 Study processing

### 2.1 Research tools and methodology

This study employs CiteSpace (version 6.3. R1), a visualization software based on citation analysis theory, to conduct bibliometric analysis of MLD. Through its pathfinding network algorithms, CiteSpace effectively identifies knowledge foundations, research hotspots, and evolutionary trends in the field (Li and Chen, 2022). The software enables comprehensive visualization of the research domain’s knowledge structure (Van Eck and Waltman, 2017).

The analysis incorporates three key components: (1) author and document co-citation analysis to establish the knowledge base of MLD research; (2) keyword clustering to identify research hotspots; and (3) temporal analysis of burst terms, time-zone variations, and keyword citation networks to reveal developmental trajectories. Parameter settings include: 2000–2024 time span, 1-year time slices, and Top 50 selection per slice for co-occurrence analysis.

### 2.2 Data sources and analysis

The data is sourced from the Science Citation Index (SCI) and the Social Sciences Citation Index (SSCI) in the Web of Science Core Collection database. The Web of Science Core Collection was selected for this study due to its rigorously curated journal content and established reputation as a benchmark for high-quality, influential research across the natural sciences, social sciences, and humanities. The field of MLD is highly interdisciplinary, spanning psychology, neuroscience, education, and psychiatry. Web of Science Core Collection database offers extensive coverage of these disciplines, providing access to core journal literature essential for tracing the development of the field.

A systematic literature search was conducted in the Web of Science Core Collection on December 19, 2024, using the search formula “TS = (mathematical learning disabilities OR mathematical learning difficulties).” The search was restricted to publications dated from January 1, 2000 to December 19, 2024, with document types limited to articles and reviews, and indexes restricted to the Science Citation Index Expanded (SCI-EXPANDED) and Social Sciences Citation Index (SSCI). This search yielded an initial pool of 727 relevant publications. After the initial search, the literature was refined in two steps to ensure relevance and precision. Duplicate publications were first removed automatically using CiteSpace. Next, two researchers independently screened the remaining titles and abstracts based on inclusion criteria requiring that each publication be an empirical study or review focused specifically on mathematical learning disabilities or difficulties, not other types of learning disorders. Book chapters, conference papers, editorials, and commentaries were excluded. Any differences in screening decisions between the researchers were discussed until agreement was reached. This process yielded a final set of 273 high quality and relevant publications for subsequent bibliometric analysis. This final collection including 158 distinct journals, 1,032 authors, 770 institutions, and spans 138 countries and regions.

**Figure 1:**
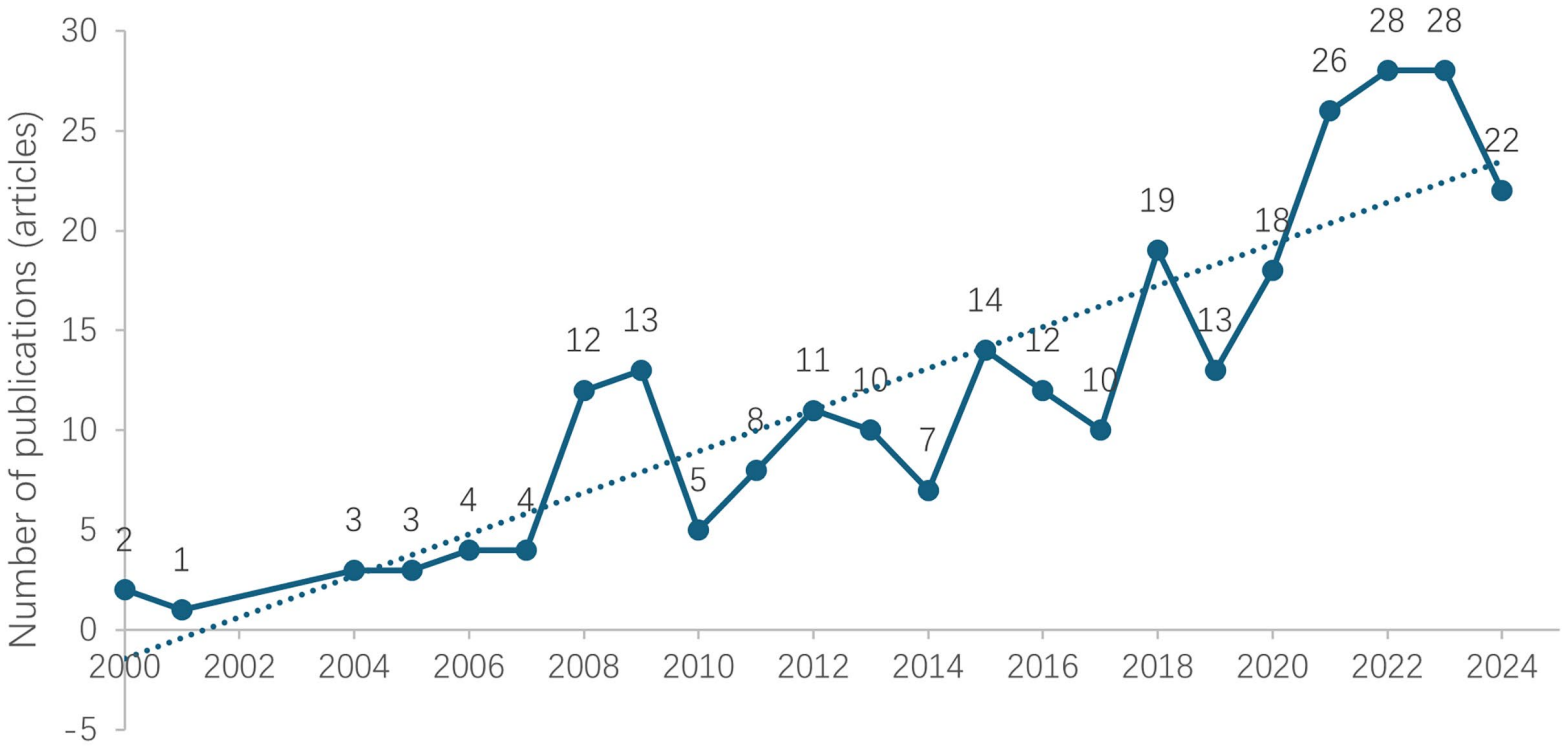
Publication trends in MLD research (2000–2024).

The annual publication data on MLD demonstrate that research in this field has attracted extensive global attention, showing an overall upward trend. As shown in Figure 1 which based on 273 source articles, the number of publications reached 13 articles in 2009, increased to 14 articles in 2015, and further rose to 19 articles in 2018. Although a minor decline was observed subsequently, the overall research output maintained a growth trend. Pay attention to 2021, since this year, publication numbers have shown significant growth, culminating in a peak of 28 publications in 2023.

From the perspective of source publication distribution, the 273 papers retrieved from WOS were published in 158 SCI and SSCI journals. The six journals with the highest number of publications and their respective counts are as follows: *DEVELOPMENTAL NEUROPSYCHOLOGY* published 12 articles, accounting for 4.4%; *PLOS ONE* published 10 articles, accounting for 3.7%; *MATHEMATICS* published 8 articles, accounting for 2.9%; *COMPUTERS & EDUCATION* published 8 articles, accounting for 2.9%; *CHILD NEUROPSYCHOLOGY* published 8 articles, accounting for 2.9%; and *FRONTIERS IN HUMAN NEUROSCIENCE* published 7 articles, accounting for 2.6%. The details are shown in Table 1.

**Table 1:** Top 6 journals by publication count.

Source	No.	Name of journal	Number	Percentage
WOS	1	DEVELOPMENTAL NEUROPSYCHOLOGY	12	4.4%
	2	PLOS ONE	10	3.7%
	3	MATHEMATICS	8	2.9%
	4	COMPUTERS and EDUCATION	8	2.9%
	5	CHILD NEUROPSYCHOLOGY	8	2.9%
	6	FRONTIERS IN HUMAN NEUROSCIENCE	7	2.6%

A content analysis of the publications reveals distinct research focuses across journals: *Developmental Neuropsychology*, *Child Neuropsychology*, and *Frontiers in Human Neuroscience* primarily investigate the neural and brain mechanisms underlying MLD; *PLOS ONE* emphasizes cognitive mechanisms associated with MLD; while *Mathematics* and *Computers & Education* concentrate on pedagogical approaches and learning processes for students with MLD. These differential research focuses not only reflect each journal’s specific academic positioning but also represent the multifaceted research directions within the MLD field, including neurobiological, cognitive, educational perspectives and so on.

## 3 Research findings

### 3.1 Intellectual foundation of MLD research

Using CiteSpace software, co-citation analysis of authors and document can be conducted. In this study, the network is built from the references of the 273 source articles, revealing the intellectual base that has shaped MLD research. The analytical results reveal the key influential scholars and highly cited references in this field, which play an important role in understanding the development of MLD research. These elements constitute the intellectual foundation of MLD studies.

#### 3.1.1 Author co-citation analysis

**Figure 2:**
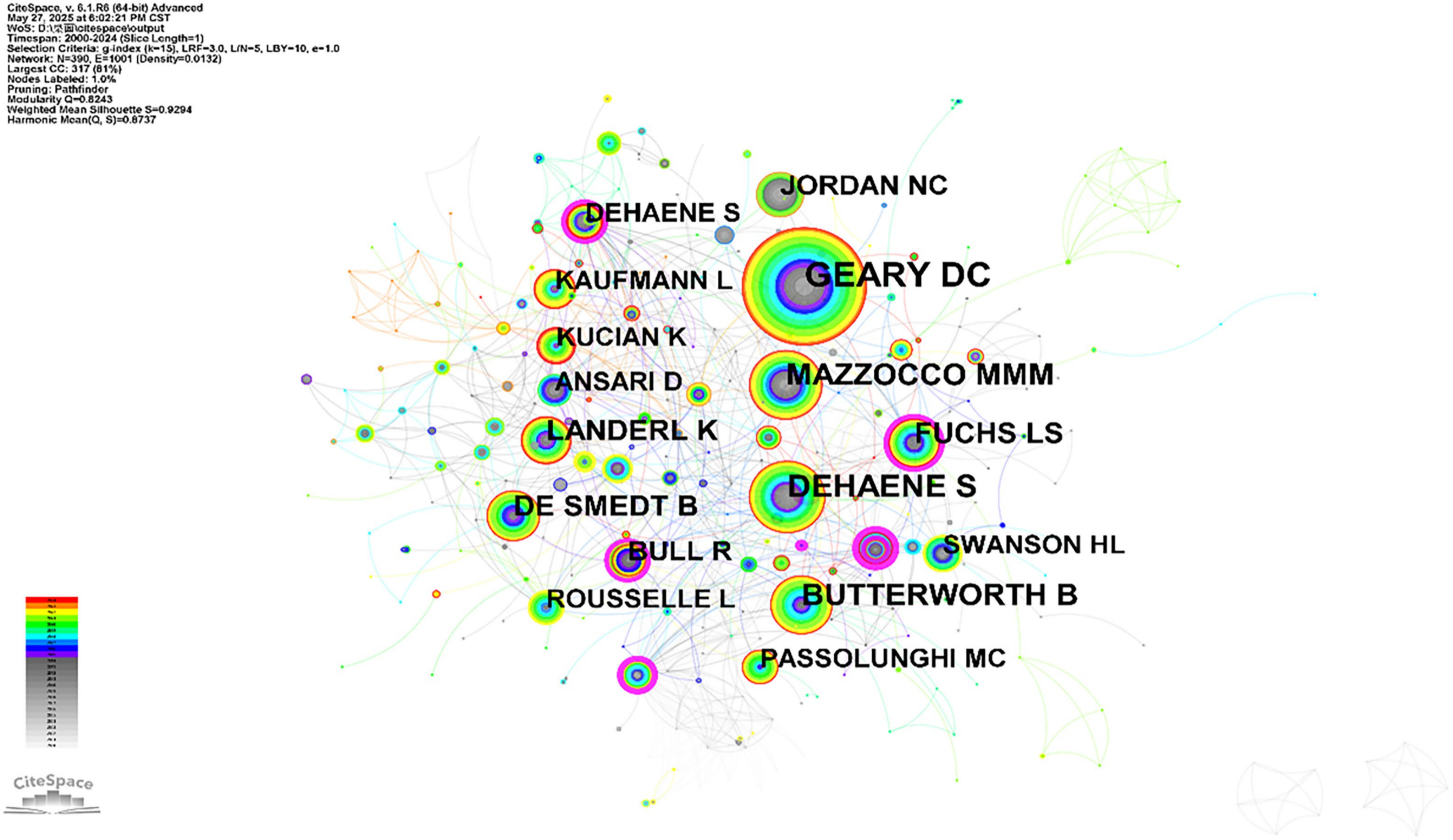
Author co-citation.

Author co-citation refers to when two or more authors are cited together across multiple publications, forming a co-citation relationship. Through author co-citation analysis, highly cited authors can be identified to determine influential researchers in the field. Furthermore, cluster analysis can be employed to examine the distribution of research topics among relevant scholars in this research domain (Li and Chen, 2022). The author co-citation map as shown in Figure 2.

The author co-citation analysis identified seven scholars with citation frequencies exceeding 40 times: Geary DC, Dehaene S, Butterworth Brian, Mazzocco Michele MM, De Smedt Bert, Jordan NC, and Landerl K.

Geary, D. C. from the Department of Psychological Sciences at the University of Missouri has achieved a co-citation frequency of 100 in mathematical learning disabilities research, with his five most influential publications being: “Mathematics and learning disabilities” (Geary, 2004), “Cognitive mechanisms underlying achievement deficits in children with mathematical learning disability” (Geary et al., 2007),” Cognitive Predictors of Achievement Growth in Mathematics: A 5-Year Longitudinal Study” (Geary, 2011a, 2011b),” Numerical and arithmetical cognition: A longitudinal study of process and concept deficits in children with learning disability” (Geary et al., 2000),” Strategy choices in simple and complex addition: Contributions of working memory and counting knowledge for children with mathematical disability” (Geary et al., 2004). The most of researches focus on cognitive mechanisms of MLD, such as working memory, counting strategies, and cognitive roots of math achievement deficits.

Dehaene, S., a researcher from Universite Paris Saclay, has a co-citation frequency of 68. His most influential works in MLD research, ranked by citation count, are: “Three parietal circuits for number processing” (Dehaene et al., 2003), “Core systems of number” (Feigenson et al., 2004), “Towards a cognitive neuroscience of consciousness: basic evidence and a workspace framework” (Dehaene and Naccache, 2001), “Experimental and Theoretical Approaches to Conscious Processing” (Dehaene and Changeux, 2011), and “Sources of mathematical thinking: Behavioral and brain-imaging evidence” (Dehaene et al., 1999). He unveiled the neural mechanisms underlying reading, mathematical calculation, and consciousness through neuroimaging techniques, proposing the neuronal recycling hypothesis.

Butterworth, B., a researcher from University College London, has a co-citation frequency of 60. His most influential works in MLD research, ranked by citation count, are: “Developmental dyscalculia and basic numerical capacities: a study of 8-9-year-old students” (Landerl et al., 2004), “The development of arithmetical abilities” (Butterworth, 2005), “Dyscalculia: From Brain to Education” (Butterworth et al., 2011) published in Science, “Number and language: how are they related?” (Gelman and Butterworth, 2005) and “Foundational numerical capacities and the origins of dyscalculia” (Butterworth, 2010). His primary contributions lie in focusing on the causes of developmental dyscalculia and basic numerical capacities, while also exploring the relationship between mathematics and language.

Mazzocco, M. M. M. from the University of Minnesota has a co-citation frequency of 55 in MLD research. Her top five most cited publications are: “Individual differences in non-verbal number acuity correlate with maths achievement” (Halberda et al., 2008), “Impaired Acuity of the Approximate Number System Underlies Mathematical Learning Disability (Dyscalculia)” (Mazzocco et al., 2011a), “Preschoolers’ Precision of the Approximate Number System Predicts Later School Mathematics Performance” (Mazzocco et al., 2011b), “Complexities in identifying and defining mathematics learning disability in the primary school-age years” (Mazzocco and Myers, 2003), and “Cognitive characteristics of children with mathematics learning disability (MLD) vary as a function of the cutoff criterion used to define MLD” (Murphy et al., 2007). Her research focused on how the acuity of the approximate number system (ANS) relates to mathematical learning disability (MLD), and also addressed its early identification and definition.

De Smedt, B. from KU Leuven (Catholic University of Leuven) has a co-citation frequency of 43. His most influential works in MLD research, ranked by citation count, are: “Associations of non-symbolic and symbolic numerical magnitude processing with mathematical competence: a meta-analysis” (Schneider et al., 2017), “The predictive value of numerical magnitude comparison for individual differences in mathematics achievement” (De Smedt et al., 2009b), “Working memory and individual differences in mathematics achievement: A longitudinal study from first grade to second grade” (De Smedt et al., 2009a), “Defective number module or impaired access? Numerical magnitude processing in first graders with mathematical difficulties” (De Smedt and Gilmore, 2011), and “Association between basic numerical abilities and mathematics achievement” (Sasanguie et al., 2012). His work focuses on numerical magnitude processing, the relationship between working memory and mathematical achievement, and the cognitive characteristics of mathematical difficulties.

Jordan, N. C. from the University of Delaware has a co-citation frequency of 42. Her most cited publications in MLD research are: “Early Math Matters: Kindergarten Number Competence and Later Mathematics Outcomes” (Jordan et al., 2009), “Early identification and interventions for students with mathematics difficulties” (Gersten et al., 2005), “Number sense growth in kindergarten: A longitudinal investigation of children at risk for mathematics difficulties” (Jordan et al., 2006), “A longitudinal study of mathematical competencies in children with specific mathematics difficulties versus children with comorbid mathematics and reading difficulties” (Jordan et al., 2003), and “The importance of number sense to mathematics achievement in first and third grades” (Jordan et al., 2010). Most of her researches focus on the development of early mathematical abilities, the early identification and intervention of mathematical difficulties, and the importance of number sense.

Landerl, K. from Macquarie University has a co-citation frequency of 40. Her most influential works in MLD research, ranked by citation count, are: “Developmental dyscalculia and basic numerical capacities: a study of 8-9-year-old students” (Landerl et al., 2004), “Comorbidity of learning disorders: prevalence and familial transmission” (Landerl and Moll, 2010), “Dyslexia and dyscalculia: Two learning disorders with different cognitive profiles” (Landerl et al., 2009), “Typical and atypical development of basic numerical skills in elementary school” (Landerl and Koelle, 2009)and “Cognitive Risk Factors for Specific Learning Disorder: Processing Speed, Temporal Processing, and Working Memory” (Moll et al., 2016). Her influential research has helped to characterize the basic numerical deficits underlying developmental dyscalculia, distinguish between the cognitive patterns of dyslexia and dyscalculia, and examine the typical and atypical development of numerical skills in children.

These seven scholars from renowned universities in Europe and the United States have established the theoretical foundation of mathematical learning disability research through their long-term and systematic studies spanning multiple dimensions. Their work addresses cognitive mechanisms such as working memory and numerical processing, neural underpinnings including parietal lobe function, as well as educational practices like early identification and intervention. Their high frequency of citations demonstrates the far-reaching influence and central importance of their contributions to this field.

#### 3.1.2 Document co-citation analysis

Document co-citation analysis refers to when two documents simultaneously appear in the references of a third article, thus establishing a co-citation relationship. By examining these co-citation relationships within a collection of literature, we perform document co-citation analysis (Li and Chen, 2022). This method enables in-depth exploration of significant literature within a research field, forming the knowledge foundation of that research domain.

This study conducted a document co-citation analysis using CiteSpace software, identifying 10 highly cited publications (co-citation frequency >6) in the field of MLD research, as detailed in Table 2. These studies, focusing on both the *neurobiological mechanisms* and *cognitive mechanisms* underlying mathematical learning disabilities, collectively form the knowledge foundation of the MLD research field.

**Table 2:** Most frequently cited literature on MLD research (2000–2024).

Co-citation count	References	Title
8	Landerl et al. (2004)	Developmental dyscalculia and basic numerical capacities: a study of 8-9-year-old students
8	Peters and De Smedt (2018)	Arithmetic in the developing brain: a review of brain imaging studies
7	Geary et al. (2004)	Mathematics and learning disabilities
7	Geary et al. (2007)	Cognitive mechanisms underlying achievement deficits in children with mathematical learning disability
7	Rivera et al. (2005)	Developmental changes in mental arithmetic: evidence for increased functional specialization in the left inferior parietal cortex
6	Arsalidou et al. (2018)	Brain areas associated with numbers and calculations in children: meta-analyses of fMRI studies
6	Bruandet et al. (2004)	A cognitive characterization of dyscalculia in Turner syndrome
6	Mazzocco et al. (2011a)	Impaired Acuity of the approximate number system underlies mathematical learning disability (Dyscalculia)
6	Price et al. (2007)	Impaired parietal magnitude processing in developmental dyscalculia
6	Rotzer et al. (2008)	Optimized voxel-based morphometry in children with developmental dyscalculia

##### 3.1.2.1 Research on the neurobiological mechanisms of MLD

Peters and De Smedt in their article *“Arithmetic in the Developing Brain: A Review of Brain Imaging Studies,”* demonstrated through neuroimaging studies that the development of arithmetic skills, which important for children’s mathematical learning, is associated with neural networks involving the prefrontal cortex, posterior parietal cortex, occipitotemporal regions, and hippocampus (Peters and De Smedt, 2018). In the study *“Developmental Changes in Mental Arithmetic: Evidence for Increased Functional Specialization in the Left Inferior Parietal Cortex.,”* researchers investigated neurodevelopmental trajectories of mental arithmetic using participants aged 8–19 years. Their neuroimaging results revealed distinct age-related activation patterns: older adolescents exhibited significantly stronger activation in the left parietal cortex (particularly along the supramarginal gyrus and adjacent anterior intraparietal sulcus) and left occipitotemporal cortex. In contrast, younger participants showed predominant activation in prefrontal regions (including both dorsolateral and ventrolateral prefrontal cortex) and the anterior cingulate cortex, indicating their greater reliance on working memory and attentional resources to achieve comparable arithmetic performance. Additionally, younger subjects demonstrated heightened activation in the hippocampus and dorsal basal ganglia, reflecting their increased dependence on both declarative and procedural memory systems during arithmetic processing (Rivera et al., 2005). In the meta-analytic study *“Brain Areas Associated with Numbers and Calculations in Children: Meta-Analyses of fMRI Studies,”* Arsalidou et al. systematically examined fMRI data from children under 14 years old to develop a neuropsychological model of mathematical functioning. The results showed that there was activity in the parietal (e.g., inferior parietal and precuneus) and frontal (e.g., superior frontal gyrus and medial frontal gyrus) cortices, the core areas associated with mental arithmetic, as well as in brain regions not usually considered as part of the model for answering mathematical questions, such as the insula and the thalamus (Arsalidou et al., 2018). In the Article *“Impaired Parietal Magnitude Processing in Developmental Dyscalculia,”* Price demonstrated that children with pure developmental dyscalculia exhibit significantly reduced modulation of the right intraparietal sulcus in response to numerical processing demands compared to typically developing children. Their findings provide compelling evidence for a strong association between parietal lobe dysfunction and developmental dyscalculia (Price et al., 2007). Rotzer conducted a study titled *“Optimized voxel-based morphometry in children with developmental dyscalculia”* involving 12 children with developmental dyscalculia. Using voxel-based morphometry analysis, the results revealed significant structural abnormalities in the brains of these children. Specifically, they exhibited markedly reduced gray matter volume in the right intraparietal sulcus, anterior cingulate cortex, left inferior frontal gyrus, and bilateral middle frontal gyri. Regarding white matter, these children also showed significantly smaller volumes in the left frontal lobe and right parahippocampal gyrus. The reduction in gray and white matter volumes, particularly within the frontoparietal network, may underlie their impaired arithmetic processing abilities. Additionally, the decreased white matter volume in the parahippocampal gyrus could adversely affect fact retrieval and spatial memory processing (Rotzer et al., 2008).

Taken together, these studies help build a multidimensional picture of how the brain works in MLD, though their methods and findings still need further examination. The review by Peters and De Smedt (2018) clearly shows that learning arithmetic involves a network of brain regions including the prefrontal and parietal cortices and the hippocampus, but it does not fully explain how these areas work together dynamically. Rivera et al. (2005) looked at how mental calculation changes with age and found that older teens rely more on parietal areas, while younger children use more prefrontal regions, suggesting greater dependence on attention and working memory. Although insightful, the study’s cross-sectional design makes it hard to draw firm conclusions about development over time. Arsalidou et al. (2018) combined results from multiple brain imaging studies and showed that math tasks activate not only classic math-related areas like the parietal and frontal lobes, but also other regions such as the insula and thalamus. Still, this expanded model needs to be confirmed by more research. Some of the most direct evidence comes from Price et al. (2007) and Rotzer et al. (2008), who found that children with dyscalculia show both functional and structural differences in the parietal lobe, especially in the right intraparietal sulcus. However, Rotzer’s study included only 12 children, making it difficult to generalize the results.

In summary, while these studies highlight the neurobiological mechanisms of MLD, more work is needed with larger groups, longer-term designs, and diverse methods to better understand how different brain regions contribute to math difficulties and how they affect learning.

##### 3.1.2.2 Research on cognitive mechanisms of MLD

Landerl investigated children with dyscalculia, reading difficulties, or both in their study *“Developmental dyscalculia and basic numerical capacities: a study of 8-9-year-old students.”* The results demonstrated that dyscalculia represents a specific deficit in basic numerical processing, rather than a consequence of other cognitive impairments (Landerl et al., 2004). Geary DC analyzed fourth-grade mathematics achievement data in their study *“Mathematics and learning disabilities,”* revealing that children with MLD relied on string retrieval when performing addition tasks. This finding suggests impaired inhibitory control over irrelevant information during fact retrieval in these children (Geary, 2004). Furthermore, Geary DC examined cognitive deficits in MLD in their study *“Cognitive mechanisms underlying achievement deficits in children with mathematical learning disability.”* They found that children with MLD exhibited impairments across various cognitive math tasks, with most deficits being mediated by either working memory or processing speed (Geary et al., 2007). Bruandet identified cognitive impairments in Turner syndrome patients in their study *“A cognitive characterization of dyscalculia in Turner syndrome.”* The researchers found these individuals (a genetic condition in females occurring in 1 in 2,500 births, characterized by partial or complete absence of one X chromosome) exhibited deficits in cognitive estimation, rapid counting, and arithmetic processing (Bruandet et al., 2004). Mazzocco MMM demonstrated in their study *“Impaired Acuity of the Approximate Number System Underlies Mathematical Learning Disability (Dyscalculia)”* that deficits in the Approximate Number System (ANS) represent a specific impairment in students with MLD. The findings revealed significantly lower ANS acuity in these students compared to typically developing peers (Mazzocco et al., 2011a).

The studies discussed above explore various cognitive mechanisms behind MLD, yet they also reveal ongoing theoretical debates and methodological limitations. Landerl et al. (2004) laid important groundwork by showing through group comparisons that dyscalculia is a distinct impairment, not just a result of broader cognitive deficits. However, whether these findings apply to all types of MLD remains unclear. Geary’s studies (2004, 2007) systematically demonstrated the central role of working memory and processing speed in MLD, shifting attention from domain-specific deficits to the importance of general cognitive resources. This helped better explain the varied nature of MLD. That said, these conclusions are largely based on correlational data, more longitudinal studies are needed to establish cause and effect. Overall, it appears MLD does not have a single cognitive cause. Instead, it may stem from problems in one or more pathways, such as number sense, working memory or how these interact. Future studies should use more refined measures, include larger and more diverse groups, and work toward integrating different theories to clarify how these cognitive mechanisms interact and contribute to MLD.

Analysis of author co-citation and document co-citation networks reveals that Geary, D. C., Mazzocco, M. M. M., and Landerl, K. emerge as both highly co-cited authors and contributors of frequently co-cited publications. This pattern suggests a relatively concentrated knowledge production structure within the field of mathematical learning disabilities research.

### 3.2 Research hotspots in MLD

Analysis of keywords within a research field can reveal its hotspots. This study employed CiteSpace to conduct keyword co-occurrence analysis (K = 15) on the collected literature based on the final set of 273 articles. Building upon keyword co-occurrence, cluster analysis was performed, where keyword nodes represent frequency of occurrence and connecting lines indicate co-occurrence relationships. Modularity (Q) and Mean Silhouette (S) serve as key metrics for evaluating clustering quality. A Q value closer to 1 indicates clearer cluster distribution (typically >0.3 is acceptable), while an S value closer to 1 suggests higher within-cluster consistency (typically >0.7 indicates high-quality clustering).

**Figure 3:**
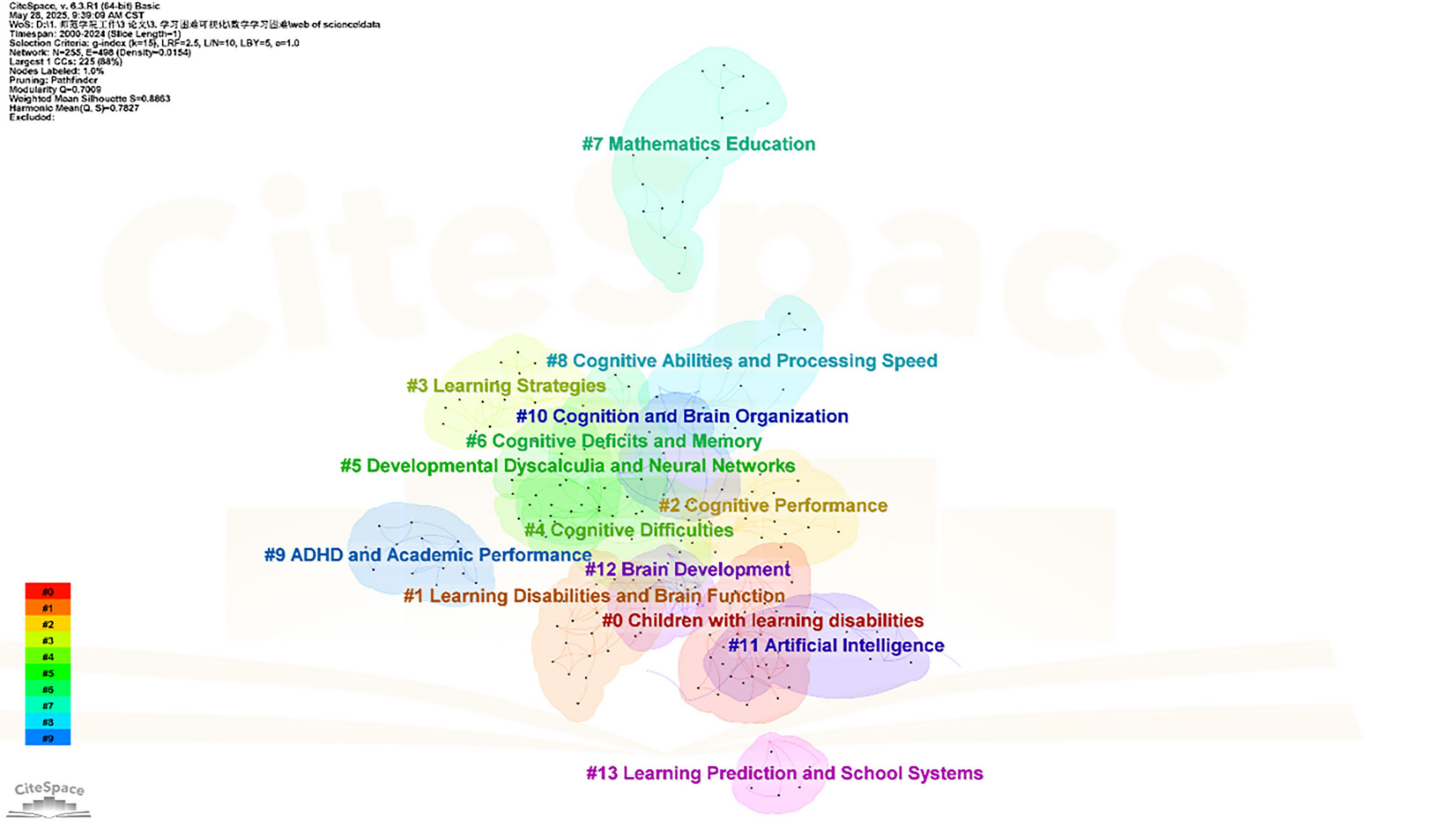
Keyword clustering.

This study through keyword clustering analysis generated 35 clusters, comprising 255 keyword nodes and 743 connecting links. The modularity Q value reached 0.7009 with a mean silhouette S value of 0.7827, indicating excellent network independence and internal consistency. Focusing on the top 14 clusters (as shown in Figure 3), we systematically analyzed their research contents and high-frequency keywords to identify four major research themes in MLD from 2000 to 2024.

The keyword cluster map (Figure 3) visually represents the intellectual structure of MLD research. The clear grouping of clusters, supported by high modularity (Q) and silhouette (S) values, indicates well-defined research clusters. Through a careful review of the literature within the relevant clusters, we found that these 14 clusters can be synthesized into four major themes. The composition of clusters #0 (children with learning disabilities), #2 (cognitive performance), #4 (cognitive difficulties), and #6 (cognitive deficits and memory) strongly indicates a cohesive research domain centered on Mathematical Disability and Cognitive Mechanisms. Similarly, clusters #1 (Learning Disabilities and Brain Function), #5 (Developmental Dyscalculia and Neural Networks), #10 (Cognition and Brain Organization), and #12 (Brain Development) collectively reflect a research domain focused on Neural Mechanisms. Clusters #3 (Learning Strategies), #8 (Cognitive Abilities and Processing Speed), #9 (ADHD and Academic Performance), and #13 (Learning Prediction and School Systems) together suggest a cohesive theme around Cognitive, Affective, and Sociocultural Factors. Finally, clusters #7 (Mathematics Education) and #11 (Artificial Intelligence) form a research domain emphasizing Educational Strategies, as further detailed in Table 3.

**Table 3:** Research clusters and hotspot topics in MLD (2000–2024).

Theme	Cluter	Label	Keyword nodes	S value	High-frequency keywords
Mathematical disability and cognitive mechanisms	0	Children with learning disabilities	29	0.919	Children, disability, learning disability, activation, dyscalculia, numerical capacity
	2	Cognitive performance	20	0.88	Difficulty, attention, cognition, performance, mathematical learning disabilities
	4	Cognitive difficulties	19	0.772	Brain, acuity, approximate number system, numerical cognition, memory, mathematics difficulty, angular gyrus
	6	Cognitive deficits and memory	16	0.842	Deficits, working memory, arithmetical cognition, turner syndrome, mathematical learning disability
Neural mechanisms	1	Learning disabilities and brain function	23	0.787	Learning disabilities, meta-analysis, brain activation, executive functions, language, language
	5	Developmental dyscalculia and neural networks	19	0.987	Developmental dyscalculia, adolescents, number, skills, mathematics, neural networks, cognitive phenotype
	10	Cognition and brain organization	12	0.78	Short term memory, intraparietal sulcus, math, multiplication, brain organization, hyper connectivity, mathematical cognition
	12	Brain development	10	0.852	Dyslexia, individual differences, representation, brain development, brain imaging, brain training
Cognitive, affective, and sociocultural factors	3	Learning strategies	19	0.921	Academic achievement, anxiety, ability, mathematical knowledge, strategy
	8	Cognitive abilities and processing speed	14	0.836	Achievement, cognitive addition, capacity, processing differences, processing speed, computation
	9	ADHD and academic performance	13	0.952	ADHD, academic performance, attention deficit/hyperactivity disorder, intelligence
	13	Learning prediction and school systems	5	0.99	Predictors, school, systems, Chinese, associative learning
Educational strategies	7	Mathematics education	16	1	Students, mathematical models, algorithm, assistive technology, education, reinforcement learning
	11	Artificial intelligence	10	0.976	Artificial neural network, machine learning, mathematical model, growth, kindergarten

The four major research themes identified through keyword clustering are intrinsically built upon the intellectual foundation of the field, as revealed by the co-citation analysis of influential publications in Section 3.1. The following subsections will explore each theme in detail, explicitly linking the current research fronts to their foundational knowledge base.

#### 3.2.1 Mathematical disability and cognitive mechanisms

Through comprehensive analysis and integration, this theme comprises four clusters focusing on research regarding the characteristics of MLD and their cognitive mechanisms. The content encompasses not only the core deficits observed in students with MLD but also explores the etiology of these challenges from cognitive capability perspectives. Studies within this cluster primarily employ methodologies including literature reviews, meta-analyses, and large-scale surveys. This theme represents a central subject in MLD research. As previously discussed in Section 3.1.2, which covers foundational literature on the cognitive mechanisms of MLD, there also exist other influential studies and viewpoints within this research focus that are worth further discussion.

Regarding the typical characteristics and core deficits of MLD, there are different voices in the research field. Geary et al. (2008) explored the developmental characteristics of number line representation in children with MLD. The study found that children with MLD performed significantly worse than typically developing children in number magnitude comparison and number line estimation tasks. Their deficits in quantitative representation were closely related to working memory and symbolic number processing abilities. Furthermore, in a later review, Geary (2011b) summarized findings on children with persistently low mathematical achievement to outline their characteristics and provide theoretical support for developing cognitive interventions. The review identified specific cognitive features associated with these difficulties, indicating that deficits in understanding numerical magnitude, retrieving arithmetic facts, and learning mathematical procedures are core characteristics of mathematics learning difficulties. Mammarella et al. (2021) noted in their review article that two main hypotheses exist in the field of MLD research: the domain-specific core deficit hypothesis (represented by Butterworth, Dehaene, and Piazza) and the domain-general hypothesis (championed by Geary and Passolunghi). However, their large-scale study involving 1,303 children found no specific core deficit characterizing MLD. In recent years, research on MLD has shifted increasing attention toward the domain-general hypothesis, exploring the role of domain-general cognitive skills in mathematical learning (Agostini et al., 2022).

Regarding the cognitive mechanism of MLD, Karagiannakis at 2014 proposed a classification framework of learning difficulties in mathematics based on cognitive functions. The research holds that various cognitive functions such as quantitative processing ability and working memory all play important roles in mathematics learning. This framework provides a strong theoretical basis for the early diagnosis and personalized intervention of MLD (Karagiannakis et al., 2014). Agostini et al. (2022) conducted a PRISMA systematic literature review analyzing 46 studies comparing children with mathematical difficulties and typically developing children across domain-general cognitive domains, including processing speed, executive functions, attention, short-term and long-term memory, and phonological awareness. Phonological awareness refers to the metalinguistic skill of perceiving and manipulating the sound structures of spoken language. It is considered a domain-general skill because it supports the formation of phonological representations in memory, thereby facilitating the acquisition of number words and arithmetic facts. The analysis revealed that deficits in executive functions, processing speed, and attention directly impacted mathematical performance, while point out that the influence of long-term memory and language awareness on mathematics learning requires further attention from researchers.

#### 3.2.2 Neural mechanisms of MLD

As outlined in the preceding document co-citation analysis (Section 3.1.2), ten highly cited publications were identified and categorized into two primary research domains: the neurobiological mechanisms of MLD and the cognitive mechanisms of MLD. In contrast, the current section (Section 3.2.2) originates from a keyword clustering analysis that revealed 14 major research hotspots, which were further synthesized into four broader themes, one of which is termed “Neural Mechanisms of MLD.” While the titles of these sections may appear similar, their underlying logic and analytical focus differ significantly. Section 3.1.2 examines the intellectual foundation of MLD research through highly cited references, whereas Section 3.2.2 emphasizes the hotspot topics under the theme of neural mechanisms, supported by representative contemporary studies.

Through comprehensive analysis and integration, this theme encompasses four clusters focusing on brain structure, neural networks, activity mechanisms, and developmental processes. This theme constitutes a central focus within MLD research. As outlined earlier in Section 3.1.2, which addresses foundational literature on the neurobiological mechanisms of MLD, there are additional influential studies and perspectives within this domain that are worth further discussion. These studies have, to some extent, further expanded the research on the neural mechanisms of MLD.

Research in this area primarily employs neuroimaging techniques such as MRI, fNIRS, and behavioral experiments to investigate the etiology, influencing factors, and neurocognitive mechanisms underlying MLD. Numerous studies have demonstrated that structural damage or functional abnormalities in specific brain regions are critical contributors to learning disabilities. As a significant subtype of learning difficulties, mathematical learning difficulties are similarly linked to structural or functional abnormal in specialized neural circuits. The following influential studies and perspectives have emerged under this research focus.

Theoretical and empirical research exploration on the relationship between MLD and neural mechanisms. Smedt employed fMRI to explore the neural correlates of single-digit arithmetic (addition and subtraction) in 10- to 12-year-old children. The study revealed that large-number problems and subtraction tasks predominantly activated the anterior parietal network (including the intraparietal sulcus), while the left hippocampus showed significant engagement during small-number and addition tasks. Furthermore, children with low arithmetic fluency exhibited heightened activation in the right intraparietal sulcus during small-problem solving, suggesting continued reliance on quantity-based strategies rather than memory retrieval. The authors proposed this pattern as a potential neural marker of retrieval deficits in children with MLD. This work elucidated the dynamic hippocampal-parietal interplay and neural substrates of strategy selection differences during arithmetic skill development (De Smedt et al., 2011). Geary focused on the neural mechanisms of early mathematical development, particularly examining the role of the Approximate Number System (ANS) which linked to children’s mathematical achievement, and proposed a dual-system model of mathematical development. This model distinguishes between the ANS (an evolutionarily conserved perceptual ability) and domain-general systems (e.g., executive control and logical reasoning), analyzing their interactions in mathematical learning from both neurobiological and evolutionary perspectives. The study emphasized that advancing understanding of neurocognitive and cognitive mechanisms underlying mathematical skill development holds significant implications for designing educational strategies and informing government policies (Geary and Moore, 2016). Fletcher and Grigorenko reviewed shifts in research paradigms over five decades from the historical perspective of learning disabilities. They highlighted how interdisciplinary advances in cognitive science, genetics, and neuroimaging have driven the evolution from static neuropsychological assessments to dynamic intervention-response evaluations. Their analysis revealed the multi-dimensional nature of MLD while underscoring the critical roles of cognitive functions and neural mechanisms in both diagnosing and intervening (Fletcher and Grigorenko, 2017).

Some studies have also noted that developmental dyscalculia is associated with neural activity in learning, working memory and language disorders. Regarding white matte aspect, early structural neuroimaging studies suggested that learning disabilities such as dyscalculia and dyslexia might be associated with differences in white matter integrity (Rotzer et al., 2008; Rykhlevskaia, 2009). However, recent findings challenge this perspective. Moreau et al. (2018) employed diffusion tensor imaging (DTI) and Bayesian analysis to compare white matter integrity in individuals with dyslexia and dyscalculia. Their results showed no significant differences in fractional anisotropy (FA) values between the two groups, questioning the notion of white matter abnormalities as a primary factor in MLD (or dyscalculia). This also suggests that previously reported stable structural white matter differences between dyslexia and dyscalculia may not be as reliable as once thought (Moreau et al., 2018). In another aspect, Nazife Ayyildiz at 2003 conducted a comparative analysis of white matter microstructure using diffusion-weighted MRI and probabilistic fiber tractography in 16 typically developing children and 10 children with developmental dyscalculia (DD). The study revealed significantly reduced white matter coherence and shortened neural fiber pathways in the left superior longitudinal/arcuate fasciculus and anterior thalamic radiation regions of children with dyscalculia. These brain areas are closely linked to memory and language functions, supporting the hypothesis of left-lateralized language-mathematical network abnormalities in developmental dyscalculia (Ayyildiz et al., 2023).

While this section overlaps in content with the preceding Section 3.1.2 to some extent, it is important to note that Section 3.1.2 established the foundational neurobiological mechanisms of MLD, whereas this section focuses on research hotspots and represents an extension of the earlier discussion. This further explore the importance of neural mechanisms in MLD research. These studies not only corroborate earlier findings but also enrich our understanding of the neural substrates underlying MLD.

#### 3.2.3 Cognitive, affective, and sociocultural factors in mathematical learning difficulties

Through integrated analysis, this topic includes four clusters, mainly involving the influencing factors of MLD, including both internal factors such as learning strategies and emotions, as well as external social and cultural factors.

Regarding internal influencing factors of MLD. Sella investigated strategy selection abilities through computational estimation tasks (e.g., two-digit addition estimation) by comparing children with ADHD (4th-5th graders) and typically developing children. The study revealed that children with ADHD exhibited significantly lower frequency of optimal strategy selection and reduced estimation accuracy compared to their peers. These findings support the view that deficits in strategy selection are a critical factor contributing to differences in mathematical performance among children (Sella et al., 2019). Moustafa further investigated the impact of math anxiety on academic performance in mathematics. From the theoretical perspectives of attentional control theory and the conflict monitoring theory, they systematically reviewed the cognitive and neural foundations of math anxiety. The study proposed an integrated network model incorporating math anxiety, cognitive processes, and brain mechanisms, which not only deepens our understanding of math anxiety mechanisms but also provides a theoretical basis for corresponding educational strategies (Moustafa et al., 2020).

Regarding external influencing factors of MLD. At first, language deprivation may impact mathematical achievement. Santos and Cordes conducted a systematic study on the development of mathematical abilities in deaf and hard-of-hearing (DHH) children. The research found that due to limited early exposure to sign language, these children exhibited significant mathematical delays beginning at age 3 compared to their hearing peers, with this disparity persisting into adulthood. Through a review of existing literature, the study proposed two mechanisms by which language deprivation may affect mathematical development in DHH children: (1) insufficient linguistic input leading to delayed early numerical concept formation and problem-solving skills, and (2) differences in working memory capacity further impairing mathematical task performance. This research highlights the crucial connection between language and mathematical cognition, providing important evidence for specialized educational strategies (Santos and Cordes, 2022). Secondly, regional and gender differences may also affect mathematical achievement. Amalina and Vidakovich conducted a cross-sectional study investigating the development of mathematical problem-solving skills among 7th to 9th-grade students (*n* = 1,067) in East Java, Indonesia. The data revealed that students’ mathematical problem-solving abilities improved significantly from 7th to 8th grade but stagnated in 9th grade, with notable urban–rural and gender disparities: urban students outperformed their rural counterparts, and female students performed better than males. The researchers suggested these differences may reflect unequal distribution of educational resources and called for greater societal attention to balanced allocation of educational opportunities (Amalina and Vidakovich, 2023).

#### 3.2.4 Educational strategies of MLD

Through integrated analysis, this theme primarily consists of two clusters: mathematics education and artificial intelligence. The content covers educational strategies and approaches for MLD, including early prediction and the educational approaches for supporting individuals with MLD.

Regarding early cognitive prediction of MLD, Traff et al. (2020) conducted a retrospective longitudinal study (kindergarten to sixth grade) that revealed significant cognitive differences between high and low-achieving mathematics students (HMA/LMA) from an early age. The study demonstrated that HMA children exhibited multiple advantages in verbal arithmetic, logical reasoning, and executive functions, while LMA children showed persistent deficits in these corresponding domains. This research highlights the predictive value of preschool cognitive characteristics for mathematical ability. Regression analysis further identified that superior performance in verbal arithmetic, logical reasoning, and executive functions is crucial for developing excellent mathematical skills.

On neuroplasticity-based educational strategies for MLD. Iuculano et al. (2015) conducted 8-week one-on-one cognitive tutoring educational strategies combined with fMRI. Their study demonstrated that cognitive tutoring not only improved mathematical performance but also normalized functional responses in the frontoparietal and ventral temporo-occipital regions. Machine learning analyses revealed that post-intervention brain activity patterns in children with MLD showed no significant differences from typically developing children, providing neural evidence for the educational strategies’ efficacy.

On practical educational strategies for MLD. Fuchs et al. (2008) focused on intensive educational strategies for third-grade students with MLD. The study proposed seven effective educational strategies principles: the first six related to the design of intensive tutoring, such as structured instruction, explicit feedback, and systematic practice, while the seventh involved ongoing progress monitoring. The results showed that although the overall educational strategies effect was significant, some students still did not reach the expected learning targets, highlighting the need to dynamically adjust educational strategies based on monitoring data. Lein et al. (2020) conducted a meta-analysis synthesizing 33 studies to systematically evaluate educational strategies effects for students with MLD. They found that educational strategies effectiveness was influenced by factors such as grade level and interveners, and noted that schema-based transfer instruction produced the best outcomes. Around the same time, Kiru et al. (2018) systematically reviewed technology-mediated mathematics (TMM) educational strategies studies and found that TMM strategies generally had a positive effect on students’ mathematical performance.

### 3.3 Evolution of research frontiers in MLD studies

The burst terms in CiteSpace can be used to analyze the frontier dynamics of a research field, with their contribution to the field reflected by burst strength. More recent years indicate current research focuses, representing the frontiers of the field. The keyword time-zone map displays the evolutionary trajectory of the research area across different time periods by accumulating the initial appearance year and subsequent frequency of keywords, thus illustrating the developmental pathway (Li and Chen, 2022).

#### 3.3.1 Burst term analysis

**Figure 4:**
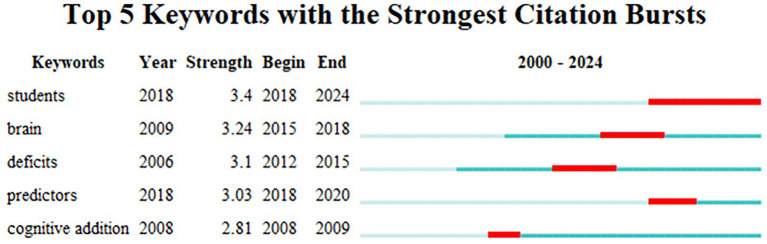
High-burst keywords in MLD research (2000–2024).

CiteSpace analysis reveals that in MLD research from 2000 to 2024, there were five high-frequency burst keywords with durations exceeding 2 years (*γ* = 0.8), which were students, brain, deficits, prediction, and cognitive enhancement. Ranked by burst strength as shown in Figure 4.

The highest burst strength keyword was “students” (burst strength = 3.4), which first appeared in 2018 and remained highly prominent through 2018–2024. This was followed by “brain” (burst strength = 3.24), initially emerging in 2009 and showing high prominence during 2015–2018. The third was “deficits” (burst strength = 3.1), first appearing in 2006 with high prominence during 2012–2015. Fourth was “predictors” (burst strength = 3.03), appearing in 2018 and remaining prominent through 2018–2020. Finally, “cognitive addition” first emerged in 2008 and was prominent during 2008–2009.

**Figure 5:**
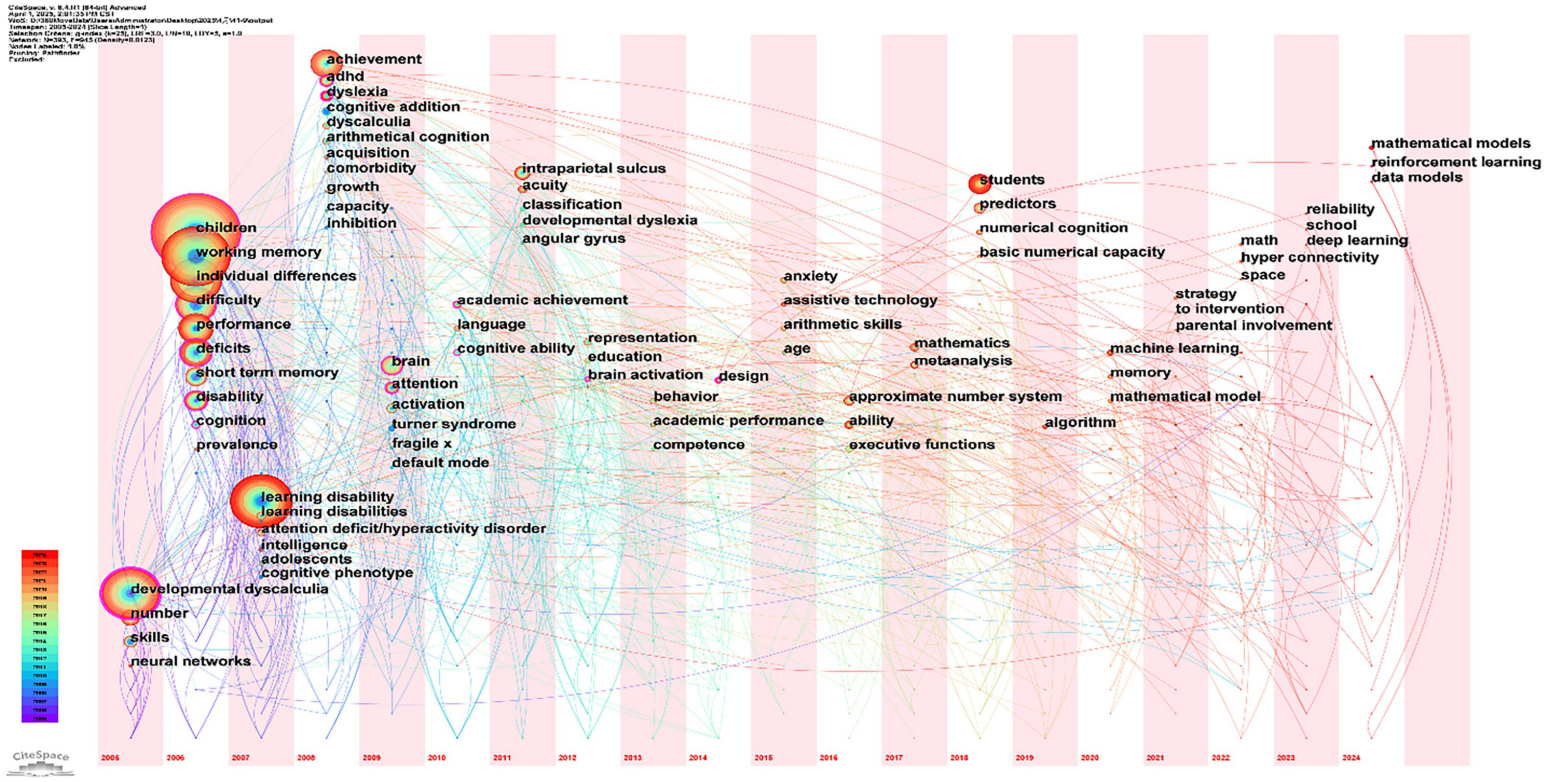
Keyword time-zone map of MLD research (2000–2024).

This clearly demonstrates that MLD research from 2000 to 2024 focused on different aspects during distinct periods. During 2008–2009, the burst keyword “cognitive addition” indicated research emphasis on cognitive functions. From 2012 to 2018, the emergence of “deficits” (2012–2015) and “brain” (2015–2018) as burst keywords reflected a shift toward investigating neural mechanisms. The 2018–2020 period saw “predictors” as the burst keyword, marking a research focus on analyzing influencing factors to estimate the probability of MLD and provide evidence for early intervention. Since 2018, the sustained burst keyword “students” (2018–2024) reveals a gradual transition from theoretical research to practical applications, with studies increasingly concentrating on student populations. Furthermore, Figure 5 clearly shows that all burst keywords continued influencing the development of this research field even after their active periods ended.

#### 3.3.2 Time-zone map analysis

Time-zone maps visualize the knowledge evolution within a research domain across temporal dimensions, aiding in understanding research themes and shifts in MLD during distinct periods. Nodes are positioned along a timeline (horizontal axis), with keywords labeled at their initial emergence year. Node size corresponds to keyword frequency across all years. The concentration of keywords in specific time-zones reflects research productivity peaks (i.e., “prosperous periods”). Co-occurrence links between keywords indicate their joint appearance in publications (Li and Chen, 2022). Figure 5 presents the keyword time-zone map of MLD research spanning 2000 to 2024.

The keyword time-zone map (Figure 5) offers a dynamic view of the field’s evolution. The left-to-right progression of keywords is not random; it tells a clear story of shifting paradigms.

As shown in Figure 5, the concentration of nodes on the left (2000–2008) around cognitive terms such as *developmental dyscalculia*, *working memory*, *short-term memory*, *learning disabilities*, and *cognitive addition* supports our identification of this as the ‘Cognitive Mechanism Exploration’ phase. The subsequent appearance of neuro-anatomical keywords, including *brain*, *intraparietal sulcus*, and *approximate number system,* in the middle time-zone (2009–2017) highlights the field’s shift into the ‘Neural Mechanism Investigation’ phase, likely influenced by progress in neuroimaging technology. Finally, the emergence of applied and contextual terms on the far right (2018–2024), such as *students*, *prediction*, *strategies*, *schools*, and *deep learning*, confirms the recent transition toward application and personalized intervention, termed here the ‘Theory-to-Practice Transition’ phase. Node size analysis shows that high-frequency keywords, including *developmental dyscalculia*, *working memory*, *children*, *learning disabilities*, and *achievement*, maintained research prominence through 2024, as indicated by their color gradients from purple to red. The concentrated emergence of new keywords between 2006 and 2009 suggests that this period had the highest number of publications.

#### 3.3.3 Evolution pathway analysis

Through analyzing the time-zone map and burst keywords, the evolutionary trajectory of MLD research can be categorized into three distinct phases: the cognitive mechanism exploration phase (2000–2008), the neural mechanism investigation phase (2009–2017), and the theory-to-practice transformation phase (2018-present).

##### 3.3.3.1 Phase 1: cognitive mechanism exploration (2000–2008)

This phase was characterized by influential keywords including *developmental dyscalculia*, *working memory*, *short-term memory*, *learning disabilities*, and *cognitive addition*, with the latter emerging as the burst keyword in 2008. Analysis of seminal publications reveals this period primarily investigated cognitive manifestations and mechanisms underlying mathematical learning difficulties. For instance, Wu employed behavioral experiments and standardized assessment tools to examine strategy use and working memory in early mental arithmetic performance. Their study established a critical relationship between children’s strategy selection in arithmetic tasks and working memory capacity, demonstrating working memory’s essential role in strategy implementation—particularly during complex computational tasks (Wu et al., 2008). This work highlighted the connection between working memory and mathematical strategy use, laying groundwork for subsequent educational strategies research.

##### 3.3.3.2 Phase 2: neural mechanism investigation (2009–2017)

This phase featured influential keywords including *brain*, *intraparietal sulcus*, and *approximate number system*, with burst keywords “deficits” and “brain.” Extensive neuroimaging studies during this period revealed structural (e.g., parietal cortex thickness) and functional (e.g., prefrontal activation patterns) abnormalities in children with mathematical learning difficulties. The research focusses consequently shifted from cognitive-behavioral aspects to systematic exploration of neural substrates. Key studies demonstrated this transition, such as Rotzer et al. (2009) identified abnormal neural network functioning in spatial working memory among children with developmental dyscalculia using fMRI, particularly highlighting under activation in the intraparietal sulcus and prefrontal cortex(Rotzer et al., 2009). Rykhlevskaia combined morphometry and fiber tracking to reveal reduced grey matter volume in parietal cortex and impaired white matter integrity in affected children (Rykhlevskaia, 2009). Ullman employed longitudinal MRI and neuropsychological assessments to demonstrate that neonatal brain abnormalities predicted childhood cognitive deficits, especially in working memory and executive function (Ullman et al., 2015). This period coincided with rapid advancements in neuropsychology and widespread adoption of neuroimaging techniques (fMRI/DTI) (Fletcher and Grigorenko, 2017), which fundamentally enabled mechanistic investigations of mathematical learning difficulties.

##### 3.3.3.3 Phase 3: theory-to-practice transition (2018-present)

This phase features newly emerged keywords including *students*, *prediction*, *strategies*, *schools*, and *deep learning*, with burst terms “students” and “prediction.” Analysis of seminal studies reveals a distinct shift from theoretical research to practical applications, focusing on specific student populations and transitioning from investigating neurocognitive mechanisms to developing educational strategies and approaches. Nelwan demonstrated through randomized controlled trials that professional coaching significantly enhances transfer effects of visual working memory training to mathematical skills (Nelwan et al., 2018), validating the critical role of human educational strategies in cognitive training programs. Hidayat employed structural equation modeling to reveal differential impacts of achievement goals, where mastery goals improved mathematical modeling via metacognitive strategies while performance goals showed detrimental effects (Hidayat et al., 2018). Benavides-Varela meta-analysis confirmed digital educational approaches (educational software/gamified platforms) significantly improved calculation skills in children with MLD (Benavides-Varela et al., 2020).

Furthermore, the connecting lines in the time-zone map indicate co-occurrence of keywords within the same publications. Figure 5 reveals strong interconnections among keywords across different research phases, demonstrating that while MLD research has transitioned toward practical applications, it maintains continuous and in-depth exploration of both cognitive and neural mechanisms. These connections suggest that although recent research emphasizes applied studies, its theoretical foundation remains rooted in earlier cognitive and neuroscientific investigations, with theoretical frameworks being continually refined through feedback from practical applications.

## 4 Conclusions and discussion

The visualization analysis of SCI and SSCI literature on MLD (2000–2024) using CiteSpace reveals the knowledge base and research hotspots in this field. Through in-depth examination of its intrinsic developmental patterns and evolutionary pathways, this study enhances understanding of MLD and explores potential future research directions.

### 4.1 The international academic community has maintained a sustained growth trend in research on MLD

In terms of publication volume, international academic research on MLD has shown a growing trend. According to the trend chart (Figure 2), the number of publications in this field is expected to continue growing steadily in the future. The temporal distribution of publications clearly demonstrates that research on MLD has been increasing in a spiral pattern since 2000, with some annual fluctuations but an overall sustained growth trend, particularly reaching its peak in 2022 and 2023. It should be noted that there were minor peaks in publication output during 2008–2009, 2018, and 2021–2023.

Detailed analysis suggests that the 2008–2009 increase may be related to the rapid development of cognitive neuroscience during that period, as researchers began focusing on the cognitive neural mechanisms of learning disabilities (Fletcher and Grigorenko, 2017). As an important research area within learning disabilities, the cognitive processing and neural mechanisms of MLD naturally became research hotspots (Nieder and Dehaene, 2009; Rykhlevskaia, 2009). The 2018 peak may have resulted from educational policy initiatives, as countries strengthened their focus on special educational needs (Gubbels et al., 2018; Knackstedt et al., 2018; Orim, 2018; Roleska et al., 2018; Wahlstrom et al., 2018), which indirectly promoted related research publications. The upward trend from 2021 to 2023 might be attributed to the impact of the COVID-19 pandemic, as remote online learning revealed MLD among more students, thereby stimulating research on corresponding educational strategies and digital approaches (Bouck et al., 2022; Enders and Kostewicz, 2023; Park et al., 2022; Satsangi et al., 2021; Shin et al., 2023).

### 4.2 Key authors have established the knowledge foundation of MLD

Analysis of highly co-cited authors and literature reveals that the fundamental knowledge base for MLD research has been established through the collective contributions of a broad international community of scholars. Prominent among these are influential researchers such as Geary, D. C. and Mazzocco, M. M. (United States), Dehaene, S. (France), and Landerl, K. (Australia), alongside other key figures like Butterworth, B., De Smedt, B., and Jordan, N. C., who have significantly advanced our understanding of mathematical cognition and learning disabilities.

An examination of highly cited authors and literatures reveal that influential researchers predominantly originate from developed Western nations, a pattern consistent with broader scientific disciplines. Historically, scholars from developed countries have maintained central positions in knowledge production, while those from less developed regions remain relatively peripheral. This gap exists because developed countries have better research infrastructure, funding allocations, and theoretical accumulation. Similar, the foundational research in cognitive science and neuroscience that supports MLD studies is predominantly conducted by institutions located in these developed regions.

These foundational studies have established the theoretical framework for MLD primarily through three aspects: cognitive-neural mechanisms, influencing factors, and interventions. Geary’s team, through a 20-year longitudinal study, identified specific deficits in core cognitive domains, including number representation, working memory, processing speed, and executive functions in children with MLD (Geary, 2004, 2011a; Geary et al., 2007). Dehaene and colleagues conducted a series of studies focusing on the cognitive neural mechanisms of conscious processing and numerical cognition. By combining behavioral experiments with neuroimaging techniques, they investigated key characteristics of conscious processing, cognitive mechanisms of number representation, and functional specialization of the parietal lobe in numerical information processing. Their work proposed the Global Neuronal Workspace (GNW) theory framework, suggesting that conscious processing relies on large-scale neural network synchronization and long-distance information integration across prefrontal and cingulate regions (Dehaene et al., 2003; Dehaene and Changeux, 2011; Dehaene and Naccache, 2001). Mazzocco’s research on early mathematical ability development established identification criteria and predictive indicators for MLD, revealing the dynamic nature of MLD definitions, subtype heterogeneity, and the association between ANS deficits and mathematical achievement. These studies demonstrated that preschoolers’ ANS could effectively predict mathematical performance in elementary school (Halberda et al., 2008; Mazzocco et al., 2011a; Murphy et al., 2007). Landerl and collaborators conducted extensive research on learning disabilities (particularly dyslexia and dyscalculia), examining their comorbidity, cognitive characteristics, and specific mechanisms. Their findings showed additive cognitive deficits in dyslexia and dyscalculia, supporting the hypothesis of separable cognitive profiles for these disorders. They also identified attention problems associated with both disorders but with different risk factor specificities, these were dyslexia linked to verbal memory and processing speed, while dyscalculia correlated with temporal processing and visuospatial memory (Landerl et al., 2009; Landerl and Koelle, 2009; Landerl and Moll, 2010; Moll et al., 2016).

These studies demonstrate remarkable interdisciplinary characteristics. By integrating methodologies from developmental psychology, cognitive neuroscience, and educational measurement, researchers have established a comprehensive evidence chain spanning from fundamental cognitive mechanisms to classroom-based interventions. It has promoted the development of the mathematics remedial teaching model based on cognitive reinforcement.

### 4.3 Research hotspots encompass both theoretical exploration and practical applications

Analysis of research hotspots in MLD reveals a balanced research landscape that integrates theoretical exploration with practical applications. Specifically, studies investigate both fundamental aspects including cognitive mechanisms (working memory, executive function, attention) and neural mechanisms (parietal cortex activation patterns, white matter structure, approximate number system), while also examining practical aspects such as sociocultural factors (family socioeconomic status, educational policies, linguistic/cultural differences) and experimental educational strategies (cognitive training, instructional adaptations, learning strategies).

According to the keyword clustering results, research hotspots from 2000 to 2024 can be categorized into 14 clusters (Table 3), including 8 clusters related to cognitive-neural mechanisms and 6 clusters concerning sociocultural factors and educational strategies. The temporal distribution of these keyword clusters (Figure 3) demonstrates an alternating pattern between cognitive-neural mechanism research and practical application studies, where practical application peaks are typically preceded by theoretical breakthroughs in cognitive-neural mechanisms. This dynamic interaction indicates MLD research consistently develops along two core dimensions, which are persistent in-depth exploration of cognitive-neural mechanisms (encompassing parietal-hippocampal coordination, white matter tract connectivity efficiency, and gene–environment interactions in the approximate number system) while continuously optimizing practical applications through enhanced interdisciplinary integration and precision strategies techniques, ultimately achieving personalized educational approaches by integrating cognitive behavioral research, neuroimaging data, and machine learning technologies.

### 4.4 “Theory-practice-theory” spiral development pathway

As a critical branch of learning disabilities research, MLD investigation initially focused on elucidating cognitive processing deficits and neurobiological abnormalities, particularly examining core cognitive domains (working memory, attention, processing speed, and executive functions) and neural substrates (approximate number system, parietal network functionality, and white matter integrity). These foundational studies established essential theoretical frameworks for understanding MLD.

With theoretical advancements, research gradually expanded to examine external influences including sociocultural factors and educational environments, while developing targeted intervention strategies. However, this expanded perspective has not diminished continued exploration of cognitive-neural mechanisms. Researchers maintain dual focus on both practical applications and fundamental mechanisms, creating a recursive “theory-practice-theory” developmental spiral, which is theoretical insights guide practical educational strategies, while strategies outcomes refine theoretical models. This iterative process has progressively constructed comprehensive mechanistic models of MLD while providing increasingly scientific foundations for early identification and precision intervention strategies. The dynamic interplay between basic cognitive-neuroscientific research and applied educational strategies studies continues to drive systematic advancement of the discipline.

In conclusion, this systematic review reveals that MLD research has yielded substantial outcomes in both theoretical frameworks and practical applications, particularly with recent breakthroughs in heterogeneity theory construction (Munez et al., 2023), technology-enhanced interventions (Islim et al., 2024; Satsangi and Raines, 2023), and comorbidity mechanism analysis (Payne and Yoon, 2024; Wakeman et al., 2023). However, several critical challenges remain: (1) Insufficient dynamic monitoring of neurodevelopmental patterns hinders the identification of MLD subtypes and their intervention trajectories; (2) Limited validation exists for the relationship between technology-based interventions’ short-term effects and cognitive improvement, with inadequate empirical evidence; (3) A comprehensive multidimensional intervention framework integrating cognitive, behavioral, and sociocultural factors is lacking. Addressing these issues may guide future research toward precise diagnosis and systematic intervention approaches, ultimately advancing MLD studies toward more integrated solutions.

## 5 Limitations and future research

This study has several limitations that should be acknowledged. Firstly, the data were drawn exclusively from the Web of Science Core Collection database. Although Web of Science Core Collection database is recognized for its rigorous selection of high-impact journals and strong coverage in mainstream English-language publications, this exclusive reliance may introduce a source selection bias. As a result, relevant literature indexed in other major databases, such as Scopus, which offers broader international coverage, ERIC, which specializes in educational research, might have been overlooked. Consequently, the findings of this bibliometric analysis may not fully capture the entirety of global research output on MLD, particularly studies published in non-English languages, regionally focused journals, or context-specific educational publications. Future studies could strengthen comprehensiveness by integrating multiple data sources, though this approach would necessitate sophisticated strategies for harmonizing coverage, standardizing citation metrics, and handling duplicates across platforms.
